# Content Analysis of Cannabis Discourses on Twitter/X in the U.S.

**DOI:** 10.1016/j.focus.2025.100408

**Published:** 2025-08-06

**Authors:** Zidian Xie, Runtao Zhou, Qihao Yun, Jianghang Wu, Zhengyuan Wang, Mengmeng Yu, Karen M. Wilson, Dongmei Li

**Affiliations:** 1 Clinical and Translational Science Institute, University of Rochester Medical Center, Rochester, New York; 2 Goergen Institute for Data Science and Artificial Intelligence, University of Rochester, Rochester, New York; 3 Department of Pediatrics, University of Rochester School of Medicine, Rochester, New York

**Keywords:** Marijuana, cannabis, Twitter/X, content analysis, deep learning

## Abstract

**Introduction:**

With the legalization of both medical and recreational cannabis use in many U.S. states, this study aims to explore public perceptions and discussions about cannabis on social media in the U.S.

**Methods:**

Twitter (now rebranded as X) data on cannabis were collected between February 2022 and February 2023 using the Twitter/X streaming Application Programming Interface. To assess the attitude of tweets toward cannabis and to determine whether Twitter/X users were cannabis users, human-guided deep-learning models called bidirectional encoder representations from transformers were used. The sex and age of users were inferred using a deep-learning facial recognition algorithm (DeepFace). The Latent Dirichlet Allocation topic model was used to comprehend the discussed topics.

**Results:**

Among 2,865,562 unique noncommercial cannabis tweets from the U.S., 648,018 tweets (22.62%) had a positive attitude toward cannabis, 234,202 (8.17%) had a negative attitude, and 1,983,342 (69.21%) had a neutral attitude. Among 821,451 unique Twitter/X users, 348,795 (42.46%) were potential cannabis users. The U.S. states allowing recreational cannabis use had 12.19 Twitter/X and cannabis users per 10,000 population, compared to 7.22 users in states without it; however, the difference was not statistically significant (*p*=0.95). The 25–34 years age group (37.14%) was the most represented among Twitter/X and cannabis users. The predominant topic in the positive tweets was “Cannabis’s medical value,” whereas the main topic in the negative tweets was “Having difficulty quitting cannabis.”

**Conclusions:**

This study provides a detailed overview of public perceptions of cannabis in the U.S., aiding policymakers and public health authorities in developing effective regulatory policies about cannabis.

## Highlights

• This study analyzed more than 2 million tweets related to cannabis from the U.S.
• This study used a deep-learning model to assess public perception of cannabis.
• Public perception of cannabis and its use varies across states with varied cannabis regulatory policies.
• Key topics were identified in cannabis-related tweets that expressed either negative or positive sentiments.

## INTRODUCTION

Cannabis, commonly known as marijuana, is a psychoactive drug regulated as a Schedule I substance under the Controlled Substances Act.^1^ Cannabis has garnered increasing attention from political candidates, state legislations, and medical researchers.^2^ As of February 2024, a total of 47 U.S. states, along with the District of Columbia (DC) and 3 territories, have legalized the medical use of cannabis.^3^ Furthermore, 24 states (such as Colorado, Washington, and California), DC, and 2 territories have legalized the recreational use of cannabis, with more states considering such actions.^4^ Despite this federal legal classification, cannabis is widely used in the U.S., with an annual use rate of 21.9%, which is 3 times higher than the global average.^5^ In 2024, approximately 55 million Americans reported currently using cannabis, and around 3,300 teenagers reported trying it for the first time every day.^6^ In addition, the average age of first-time cannabis users is generally lower than that of other illicit drug users.^7^ In 2022, for the first time in history, the total consumption of cannabis in various forms surpassed that of cigarettes.^8^

The legalization of cannabis, whether for medical or recreational purposes, not only reflects but also influences public sentiment regarding cannabis. Studies showed that residents in the states with less restrictive cannabis policies tend to have a greater number of positive discussions about cannabis on Twitter (now rebranded as X), evidenced by a high volume of favorable posts on Twitter/X.^9^, ^10^, ^11^ However, the public perception of cannabis in the U.S. is continuously evolving in response to changing policies and information.^12^ In states with medical cannabis laws, 8^th^ grade students were more likely to perceive cannabis as harmful compared to their peers in states without such laws.^12^ Attitudes toward cannabis legalization vary significantly depending on state-specific demographics and regulations.^13^ For instance, women were less likely to be aware of increased cannabis potency than men, whereas adults with a graduate degree were more likely to recognize that cannabis is becoming more potent.^13^ Understanding these trends can be immensely beneficial for policymakers and researchers.

Twitter/X is a social media platform that enables users to share their opinions and experiences, making it an ideal platform to investigate public perceptions on social issues, including cannabis use.^14^^,^^15^ Previous studies have effectively utilized Twitter/X data to examine cannabis use across various demographic and geographic groups, as well as to assess users’ sentiments.^9^^,^^16^, ^17^, ^18^ For example, 12 major topics related to cannabis were identified from cannabis-related tweets, including using cannabis and its health risks and benefits.^19^ Studies have shown that youth exposed to cannabis promotions and brands on social media had a higher likelihood of using cannabis.^20^^,^^21^ Given the rapid changes in public perception and regulatory policy regarding cannabis in the U.S., it is essential to provide an updated analysis of public sentiment and cannabis use through social media data. By analyzing social media data related to cannabis, valuable insights into the public’s beliefs and sentiments toward cannabis can be gained. This study will utilize deep-learning language models guided by the results of qualitative content analyses to more accurately assess the sentiment toward cannabis on Twitter/X.

In this study, the authors aim to understand the public perception of cannabis and its use in the U.S., particularly in relation to varying cannabis legalization policies, by analyzing Twitter/X data through natural language processing techniques and deep-learning models. By gaining insights into these perceptions, policymakers and researchers can make informed decisions regarding future regulatory policies and enhance health communications.

## METHODS

### Study Sample

This study was reviewed and approved by the Research Subjects Review Board of the Office for Human Subject Protection (STUDY00006570). Patient consent for publication is not required as the data were analyzed anonymously.

Twitter/X data on cannabis were collected through Twitter/X streaming Application Programming Interface using keywords related to cannabis or marijuana, including “marijuana,” “cannabis,” “blunt,” “bong,” “budder,” “cbd,” “ganja,” “hash,” “hemp,” “indica,” “kush,” “marihuana,” “reefer,” “sativa,” “thc,” and “weed.”^19^ Between February 26^th^, 2022 and February 11^th^, 2023, the authors collected 63,029,489 tweets related to cannabis.

As previously described, several data preprocessing steps were conducted on the collected Twitter/X dataset.^22^^,^^23^ First, based on tweet ID, the authors removed all duplicate tweets. Next, all retweets were eliminated. This study aimed to understand public perception of cannabis through analyzing noncommercial tweets. To achieve this, the authors filtered out all tweets deemed commercial or advertising tweets by using a list of keywords, such as “sale,” “offer,” and “dollar,” regardless of the type of Twitter/X user accounts.^24^ In addition, any tweets created by social bots were detected and removed with the help of the Botometer Application Programming Interface.^25^^,^^26^ Finally, tweets from the U.S. were identified based on the geolocation information provided within the tweets and Twitter/X user accounts.^27^^,^^28^ After data preprocessing, 2,865,562 unique noncommercial tweets related to cannabis from the U.S were identified.

### Measures

To assess the sentiment of tweets toward cannabis and to determine if Twitter/X users were potential cannabis users, the authors utilized a human-guided deep-learning model. The authors first randomly selected 500 cannabis-related tweets, and 5 authors labeled them (positive, negative, or neutral) according to the tweet’s sentiment toward cannabis and whether Twitter/X users were potential cannabis users (yes or no). In this study, the authors utilized a staggered pairwise hand-coding design to reduce the burden of hand-coding.^29^, ^30^, ^31^ Each human coder labeled 200 tweets, with each 100 tweets overlapping with another coder. The average Cohen’s κ statistic value among 5 different coders reached 0.63 for the sentiment and 0.78 for cannabis users, all considered substantial agreement. Any discrepancy was resolved through a group discussion among the 5 human coders, and the labeling criteria were updated accordingly. In addition, the authors did not identify any commercial tweets among these 500 noncommercial tweets. Subsequently, the authors randomly sampled another 5,000 cannabis-related tweets, and each trained author manually labeled 1,000 tweets. To further validate the consensus among human coders, 100 tweets were randomly selected from these 5,000 tweets and independently coded by 5 human coders. The Krippendorff’s α value was 0.75 for the sentiment and 0.81 for cannabis users.

To label the remaining tweets, the authors used the bidirectional encoder representations from transformers (BERT) model.^32^ As a popular state-of-the-art machine-learning model, the BERT model outperformed other traditional machine-learning text classification models.^32^ To achieve optimal efficiency and results, the pretrained BERT-large-uncased model with 24 layers and 340 million parameters was chosen. The authors customized the BERT-large-uncased model for their specific dataset by adding linear and dropout layers to ensure the correct output shape. To further optimize their model’s performance, the authors conducted hyperparameter tuning using a grid search to determine the best batch size and learning rate combination for their BERT-based model. Grid search is a systematic way of searching for the best combination of hyperparameters for a model, which is relatively straightforward to implement. After completing the grid search, the authors found that a batch size (the number of training examples processed together) of 32, a total of 15 epochs (the number of full passes through the entire training dataset), and a learning rate (the extent to which the model’s weights are updated during training) of 1e-5 produced the best results for their specific task of predicting the sentiment and classifying potential cannabis users. To train their model using their human-labeled tweets, the authors used the cross-entropy loss function, a standard function in classification tasks that measures the difference between the predicted probabilities and actual labels. In addition, the authors used the Adam optimizer, an adaptive optimization algorithm that adjusts the learning rate based on the gradient magnitudes of the parameters.

The authors divided their 5,500 human-labeled tweets into training and testing sets. The training set comprised 70% of the data, whereas the testing set contained 30%. The F1 score combines precision and recall using their harmonic mean, offering a balanced assessment of the model’s overall performance. Given the class-imbalanced nature of their labels, the authors chose to use the F1 score as the metric for measuring the model’s performance. High F1 scores were obtained by the pretrained BERT model for both cannabis user and sentiment classifications, with a score of 0.93 for classifying cannabis users and 0.84 for sentiment classification.

The authors grouped Twitter/X users into various U.S. states based on the geographical location provided by tweets or Twitter/X user accounts. For each state, the authors counted the number of unique Twitter/X and cannabis users (Twitter/X users who are potential cannabis users based on the study model). To account for variations in state population, the authors divided the number of Twitter/X and cannabis users in each state by the state population in 2022, as provided by the U.S. National Census Bureau.^33^

To estimate the age and sex of Twitter/X and potential cannabis users, the authors used a deep-learning facial recognition algorithm known as DeepFace.^34^ The user profile images were downloaded using the URLs provided in tweets. The DeepFace algorithm analyzes a user’s profile image on Twitter/X to infer their age and sex, as long as only a single face is detected in the image. Sex was classified into 2 categories: male and female. Twitter/X users were then classified into multiple age groups, including <18, 18–24, 25–34, 35–60, and >60 years.

To better understand the topics discussed in cannabis-related tweets that express either positive or negative sentiment toward cannabis, the Latent Dirichlet Allocation model was applied.^24^^,^^35^ This model identifies each topic by analyzing a distribution of words, which indicates the likelihood of that topic including those words. Based on the coherence score (the highest) and inter-topic distance (minimal overlap), the authors identified 3 major topics for tweets with positive or negative sentiment toward cannabis, respectively.

### Statistical Analysis

To compare the sentiment toward cannabis and the prevalence of potential cannabis users on Twitter/X across various U.S. states, a 2-proportion z-test was conducted at a significance level of 0.05.

## RESULTS

From February 2022 to February 2023, the authors have identified 2,865,562 unique noncommercial tweets related to cannabis from the U.S. As illustrated in Appendix Figure 1 (available online), fluctuations were observed in the number of tweets, including notable peaks on April 20, 2022 and October 6, 2022. The overall trend showed a decrease in the number of tweets mentioning cannabis during the study period.

To understand the sentiment of tweets toward cannabis, the authors used a human-guided deep-learning model to classify the sentiment of tweets as positive, negative, or neutral. Of the 2,865,562 cannabis-related tweets in the U.S., 648,018 tweets (22.62%) had a positive sentiment toward cannabis, 234,202 (8.17%) had a negative sentiment toward cannabis, and 1,983,342 (69.21%) had a neutral sentiment. A 2-proportion z-test indicated that the proportion of positive tweets was significantly higher than that of negative tweets (22.61% vs 8.17%, *p*<0.001). In addition, there was no clear temporal trend in the proportion of positive tweets throughout the study period, except for 2 notable peaks: one of them was on April 20, 2022 and the other on October 6, 2022 (Appendix Figure 2, available online).

**Figure 1:**
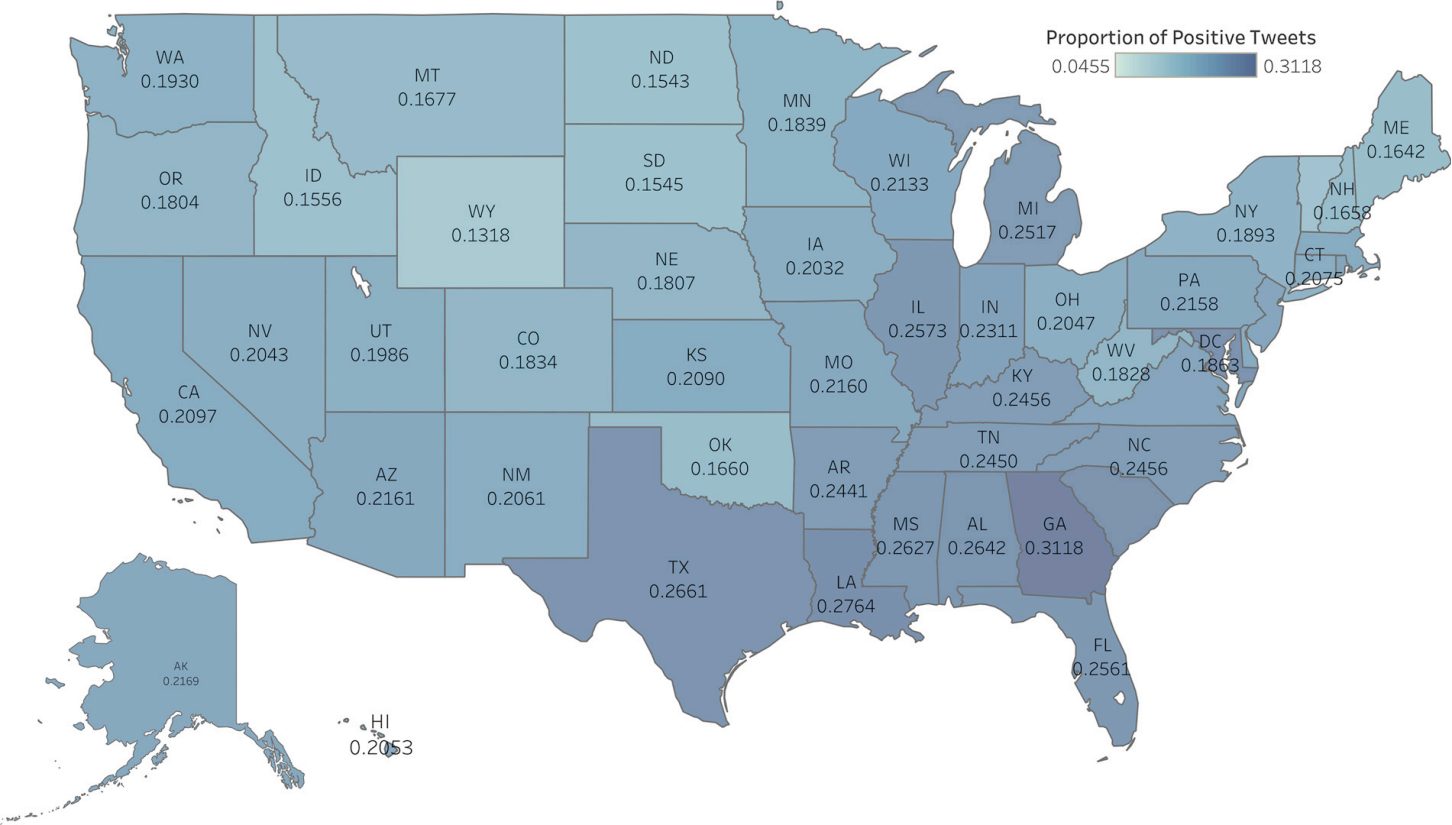
Proportion of cannabis-related tweets with a positive sentiment in the U.S.

The proportion of positive tweets varied across various U.S. states (Figure 1). Some Western states, such as Idaho, Oregon, and Wyoming, showed a relatively lower proportion of positive tweets compared to that of some Eastern states, including North Carolina, Michigan, and Georgia. Georgia had the highest proportion of tweets with a positive sentiment (31.18%), followed by Texas (26.61%) and Alabama (26.42%). In contrast, Wyoming had the lowest proportion of positive tweets (13.18%) among all U.S. states. It is well known that U.S. states have varying regulatory policies regarding cannabis. There was no significant difference in the proportion of positive tweets between states that allow recreational cannabis and those that do not (20.41% vs 21.77%, *p*=0.9045). Similarly, there was no significant difference in the proportion of positive tweets between states that allow medical cannabis and those that do not (20.89% vs 22.38%, *p*=0.9124).

From the U.S. Twitter/X data related to cannabis, 821,451 unique Twitter/X users posted cannabis-related tweets during the study period. Using a human-guided deep-learning model, the authors identified 348,795 (42.46%) Twitter/X users as potential cannabis users (i.e., Twitter/X and cannabis users). No obvious trends in the proportion of Twitter/X and cannabis users were observed throughout the study period (Appendix Figure 3, available online). Nevertheless, potential cannabis users were more likely to post positive tweets than nonusers (47.56% vs 11.60%, *p*<0.001) (Appendix Figure 4, available online). Similarly, the proportion of negative tweets among cannabis users was significantly higher than that among nonusers (9.94% vs 7.39%, *p*<0.001). In contrast, nonusers were more likely to post neutral tweets than cannabis users (81.01% vs 42.50%, *p*<0.001).

**Figure 2:**
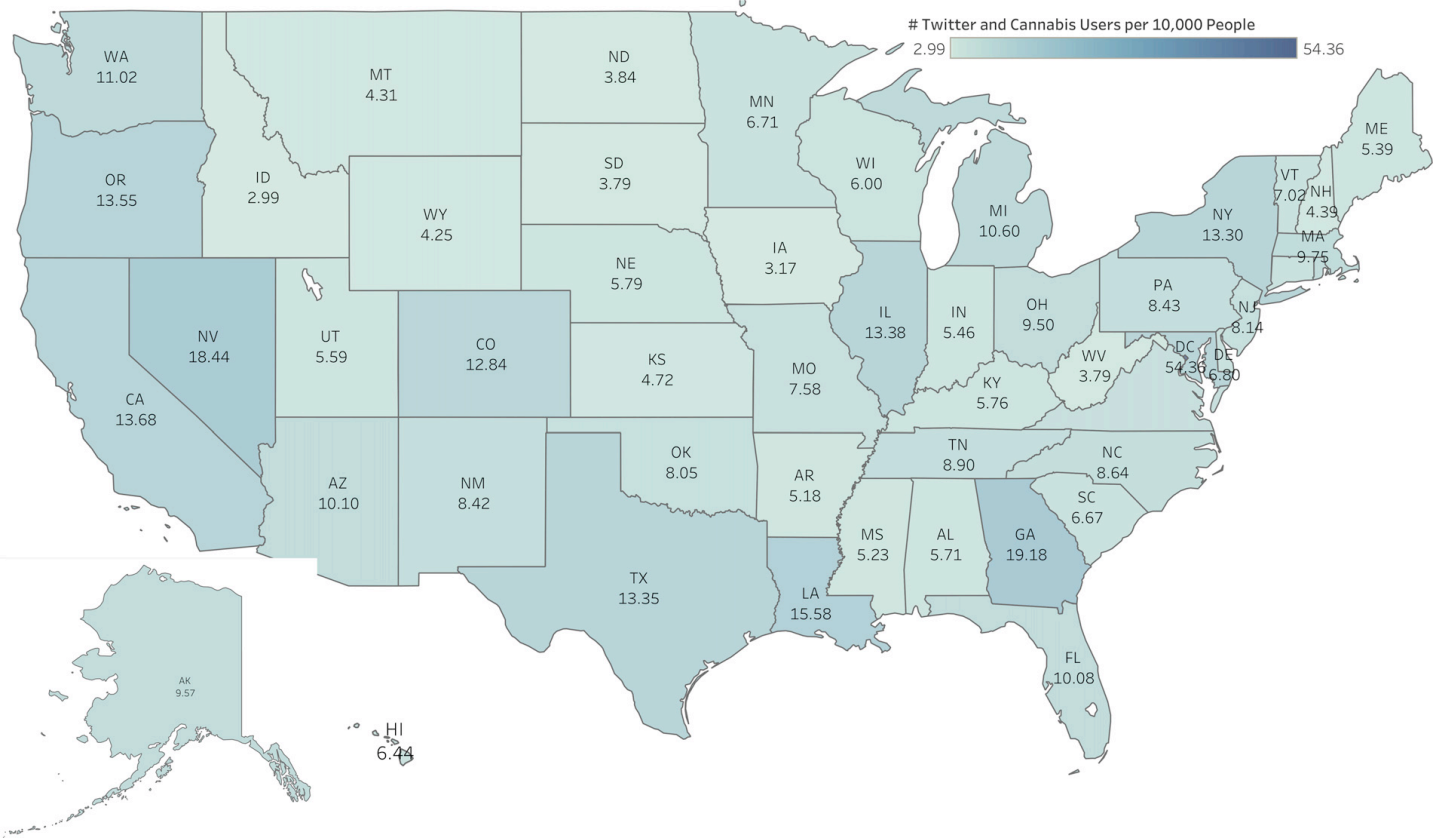
Prevalence of Twitter/X and cannabis users in the U.S.

To compare the prevalence of Twitter/X and cannabis users across various U.S. states, the authors calculated the number of Twitter/X and cannabis users per 10,000 people based on each state’s population. As shown in Figure 2, Washington DC (54.36) had the most Twitter/X and cannabis users per 10,000 people in the U.S., followed by Georgia (19.18) and Nevada (18.44). In contrast, Idaho (2.99) had the lowest number of Twitter/X and cannabis users per 10,000 people in the U.S. In states that permit medical cannabis, there was an average of 9.67 Twitter/X and cannabis users per 10,000 people, which was higher than the average of 7.81 users per 10,000 people in states without medical cannabis. However, this difference was not statistically significant (*p*=0.9840). Similarly, in states where recreational cannabis is legal, there were 12.19 Twitter/X and cannabis users per 10,000 population, whereas states without recreational cannabis had 7.22 Twitter/X and cannabis users per 10,000 people. However, this difference was also not statistically significant (*p*=0.9522).

Among the 348,795 potential cannabis users identified on Twitter/X, the authors were able to estimate the basic demographics (including age group and sex) of 26,011 Twitter/X and cannabis users (7.46%), using a face recognition algorithm (DeepFace). As shown in Appendix Figure 5 (available online), the most prevalent age group among Twitter/X and cannabis users was the 25–34 years group, comprising 37.14% of the total Twitter/X and cannabis users, followed by the 18–24 years age group (35.01%) and 35–60 years age group (24.57%). The sex distribution among the Twitter/X and cannabis users was relatively balanced, with males making up 50.69% and females accounting for 49.31%.

In the case of those tweets with a neutral sentiment, they were mainly sharing general information related to cannabis, such as the regulatory policies (e.g., Maryland bill would legalize adult-use cannabis by July 1), and asking general questions about cannabis (e.g., “looking for the best cannabis product for maximum energy, please?”). To understand why Twitter/X users showed either positive or negative sentiment toward cannabis, the authors conducted topic modeling on positive and negative tweets. As shown in Table 1, there were 3 major topics in positive tweets, including *Cannabis’s medical value* (39.8%), *Strong urge to use cannabis* (30.7%), and *Cannabis can improve overall life quality* (29.5%). In contrast, as shown in Table 2, there were 3 major topics in negative tweets against cannabis, including *Having difficulty quitting cannabis* (41.8%), *Complaining about the smell of cannabis* (30.6%), and *Worrying about the side effects of cannabis* (26.7%).

**Table 1:** Major Topics in Positive Tweets Toward Cannabis

Topics (*n*, %)	Top 10 keywords	Example tweets
Cannabis can improve overall life quality. (191,165/648,018, 29.5%)	Good, day, make, sleep, work, better, coffee, happy, always, music	• “I sleep mostly unmedicated now that am recovered from long-term psych med withdrawal (& know am autistic) but find legal (drinkable) to be a much better option.” • “Veteran with PTSD that is super happy right now. Cannabis helps me eat, sleep, and manage pain.”
Strong urge to use cannabis (198,942/648,018, 30.7%)	Need, wanna, high, smoked, gonna, roll, homie, fat, yall, hit	• “Honestly I need a fat blunt after the day I have been having.” • “I think I need a blunt of sativa every morning”
Cannabis’s medical value (257,911/648,018, 39.8%)	Legal, medical, legalize, possession, pain, really, help, love, better, lot	• “About 5 minutes ago! is my number one pain medication! I have a bad spine!” • “more dangerous prescription pain and other meds and get better relief from pain and muscle spasms. Medical cannabis also helped me when I needed strong Chemo five years ago.”

PTSD, posttraumatic stress disorder.

**Table 2:** Major Topics in Negative Tweets Toward Cannabis

Topics (*n*, %)	Top 10 keywords	Example tweets
Having difficulty quitting cannabis (97,896/234,202, 41.8%)	Alcohol, make, drugs, many, worse, brain, can’t, quit, never without	• “I wanna stop smoking marijuana but it’s so hard to quit for me.” • “I smoke marijuana about once a month. Usually on a Friday. I’ve tried so hard to quit but then the next month rolls around and I just do it.”
Complaining about the smell of cannabis (71,666/234,202, 30.6%)	Hate, stop, smell, bad, car, don’t, smelling, smoked, really, smells	• “I hate the smell of marijuana when I’m not high. It stinks.” • “It Is a nice day, and I don’t fault anyone for enjoying it however they want to enjoy it, but it smells like it was skunked six months ago.”
Worrying about the side effects of cannabis (62,532/234,202, 26.7%)	Side, effects, makes, body, getting, cancer, cause, psychosis, bad, anxiety	• “I stay away from sativa. Honestly, I don’t even think I should be smoking AT ALL but with indica I might get anxiety but with sativa it’s straight psychosis.” • “Y’all don’t realize all those drugs and alcohol come with side effects just because marijuana/percs don’t kill you straight up don’t mean long term effects don’t exist”

## DISCUSSION

In this study, the authors analyzed Twitter/X posts related to cannabis from the U.S. and showed that Twitter/X users generally expressed a more positive than negative sentiment toward cannabis. Although there was no significant temporal trend in the proportion of positive tweets, the authors observed notable differences among various U.S. states. In addition, cannabis users on Twitter/X tended to be young adults, and their prevalence varied across various states. The authors identified 3 major topics from tweets expressing either positive or negative sentiment toward cannabis.

From February 2022 to February 2023, this study found a slight decrease in the number of tweets related to cannabis. The authors identified 2 peaks in tweet activity: one of them was on April 20, 2022 and the other on October 6, 2022. April 20 is recognized as an annual unofficial holiday for a cannabis-oriented celebration.^36^ On that day, cannabis producers, consumers, and advocates come together to celebrate and smoke cannabis. On October 6, 2022, President Biden announced a pardon for certain prior federal and DC offenses for using and possessing cannabis, which generated significant public attention and discussion on Twitter/X.^37^ The temporal trends in public perception showed that tweets during these 2 peaks were generally more positive compared to other tweets, suggesting that many Twitter/X users enjoyed smoking cannabis and welcomed the pardon. Overall, these study results demonstrated that Twitter/X could effectively capture key events and regulatory changes related to cannabis.

Various U.S. states have implemented varying regulatory policies regarding cannabis, with some allowing medical use and others permitting recreational use. As of 2022, a total of 21 states (including California, New Jersey, and Arizona) permit recreational cannabis use, whereas 40 states (such as Ohio, Arizona, and California) allow medical cannabis use. Conversely, 11 states (such as Louisiana, Indiana, and Mississippi) do not permit any legal use of cannabis. In this study, analysis of Twitter/X data did not reveal any significant difference in the public perception and use of cannabis among states with varied regulatory policies. Twitter/X users who tweeted about cannabis may not represent the whole population in each state. In addition, the prevalence of Twitter/X users can vary among states, which could introduce some biases into the findings of this study. Nevertheless, the findings of this study suggest that cannabis regulations may not significantly influence public sentiment or consumption of cannabis, a finding that warrants further investigation. Similarly, using Twitter/X data from 2007 to 2019 and various sentiment analysis tools, a previous study demonstrated that the proportion of positive tweets related to cannabis did not significantly differ among states with varied regulations.^15^ A possible explanation for this observation is that the lack of regulatory enforcement and social norms over the past decade may influence public sentiment and consumption, beyond simply distinguishing between legal and illegal status. In addition, the anonymous nature of social media may provide a space to discuss stigmatized topics such as cannabis use, potentially reducing the impact of cannabis’s legal status on tweeting behavior. Another possible reason could be that the volume of online cannabis discourse may decrease in states where recreational cannabis is already legal. However, the authors did observe variations among various states. For instance, Georgia has the highest proportion of positive tweets and the highest prevalence of cannabis users based on the data of this study. Previous polls indicated that more than half of the population in Georgia supports the state government in legalizing cannabis for adult recreational use.^38^ Conversely, Idaho, Wyoming, and Montana exhibited a lower proportion of positive tweets and prevalence of cannabis users. The underlying reasons for these differences require further exploration. A previous national survey study showed that the prevalence of cannabis use was significantly higher in states with legalized recreational use than in other states.^39^ The authors observed a similar pattern, although the differences were not significant in this study. In this study, the authors used an advanced deep-learning model to estimate the demographics, including age group and gender, of cannabis users on Twitter/X. Among these cannabis and Twitter/X users, young adults aged 18–34 years were the most dominant, which aligns with previous findings.^40^^,^^41^ A possible reason for this is that there was a notable shift from the use of alcohol and other drugs to cannabis use among young adults in recent years, partially because of recreational cannabis legalization.^42^^,^^43^

Among the cannabis-related tweets, 22.61% expressed a positive sentiment toward cannabis. Topic modeling results revealed 3 main topics in this positive sentiment, such as the medical benefits of cannabis and its potential to improve overall quality of life. Cannabis is often thought to alleviate symptoms of mood or anxiety disorders.^44^ However, numerous studies have shown that regular cannabis use is associated with increased depressive, psychotic disorders, and anxiety symptoms.^45^, ^46^, ^47^ In contrast, tweets that expressed a negative sentiment primarily cited difficulties in quitting cannabis, followed by complaints about its odor and side effects. There is substantial evidence indicating that cannabis use can lead to addiction and dependence syndrome.^48^, ^49^, ^50^ The findings from this study clearly demonstrate that cannabis addiction was the primary concern mentioned in negative tweets, which warrants increased attention. Studies have indicated that exposure to cannabis-related digital content, particularly those with a positive sentiment, is positively correlated with cannabis use among youth and young adults.^21^^,^^51^ This analysis showed a greater number of positive tweets than negative ones, aligning with findings from previous studies.^52^, ^53^, ^54^ These findings suggest that the public is more inclined to share favorable opinions about cannabis on social media, which might be partially influenced by the legalization of cannabis in many U.S. states. To reduce the prevalence of cannabis use, nationwide health education campaigns, such as those conducted through social media, should be launched to inform the public about the potential health risks associated with cannabis.

### Limitations

This study has several limitations. First, the results from the Twitter/X data analysis should not be generalized to the general population. Second, many tweets lacked valid geolocation and user profile information, which were excluded from this analysis. Third, Cohen’s κ scores for human labeling were relatively low in this study. Despite the well-performing BERT models, there may have been some mislabeling in the data, which could have introduced bias. In addition, the user profile pictures used for the DeepFace model were not necessarily those of the actual Twitter/X users themselves, which could have introduced another source of inaccuracy. Fourth, because the authors did not have accurate demographic information about the Twitter/X users, they could not accurately compare public perception and cannabis use among varied demographic groups, which should be explored using other data sources in future studies. Similarly, given that the prevalence of Twitter/X use might differ among U.S. states, bias could have been introduced into the prevalence of cannabis users by normalizing it to the general population. Although there is currently no data available on the distribution of Twitter/X users by state, the findings of this study could be validated by comparing them to representative national survey data in the future. Fifth, the coronavirus disease 2019 (COVID-19) pandemic may have contributed to an increase in cannabis use.^55^ Even though this relationship could exist, the authors did not assess its impact on the current study. Sixth, the keyword list used for collecting cannabis-related tweets could have introduced bias in this study. This may occur because of missing pertinent tweets as a result of the authors not including certain relevant keywords, such as “medibles” and “420.” In addition, using ambiguous keywords such as “blunt” and “weed” could have introduced noise into the data and results. Finally, in light of the fast-evolving cannabis market and the emergence of new cannabis products (such as derived psychoactive cannabis products) in response to changing regulatory policies, it is crucial to include these novel products in the analysis and update the findings of this study with more recent data in the future.^56^ Considering the current challenges in accessing Twitter/X data after the academic account has been revoked, it is essential to explore other social media platforms including Reddit.

## CONCLUSIONS

In this study, the authors analyzed Twitter/X data with the assistance of advanced deep-learning models and showed that the public perception and use of cannabis differ across U.S. states. The authors did not observe significant differences in the public perception of cannabis among states with varying cannabis policies. Through topic modeling, the potential reasons why Twitter/X users might hold either negative or positive views toward cannabis were identified. Overall, this research provides a comprehensive understanding of the public perceptions of cannabis and the effects of varied legalization policies in the U.S., which could help inform future policy development and health communication efforts to protect public health.

## Appendix Supplementary materials

Image, application 1

## Footnotes

Supplementary material associated with this article can be found in the online version at doi:10.1016/j.focus.2025.100408.
