# Supplementary material for: Content Analysis of Cannabis Discourses on Twitter/X in the U.S
Source: AJPM Focus. 2025 Aug 6;4(6):100408. doi: 10.1016/j.focus.2025.100408 (PMC12480873; doi:10.1016/j.focus.2025.100408)
Supplement: Supplementary file 1 [file mmc1.docx]

**Appendix Figure 1.** The weekly number of tweets related to cannabis on Twitter.

**Appendix Figure 2**. The proportion of positive tweets related to cannabis over time.

**Appendix Figure 3**. The proportion of cannabis users among Twitter users.

**Appendix Figure 4**. Public perception of cannabis between cannabis users and non-users on Twitter.


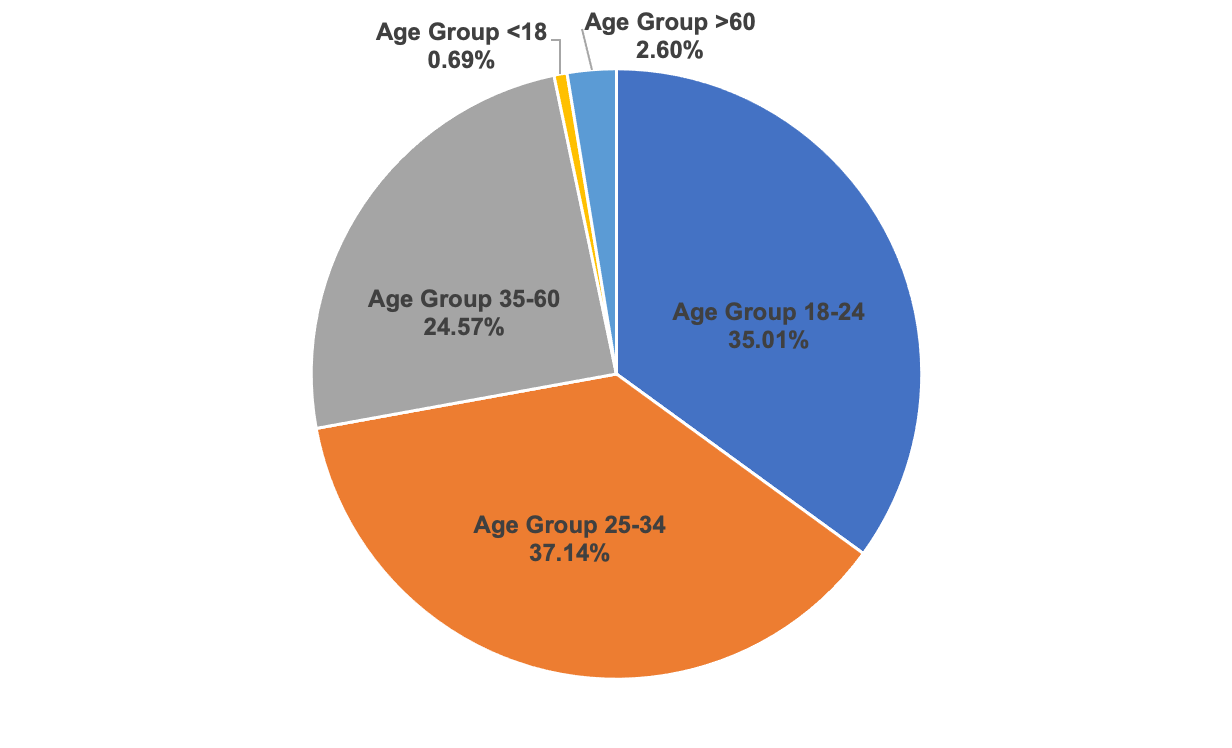

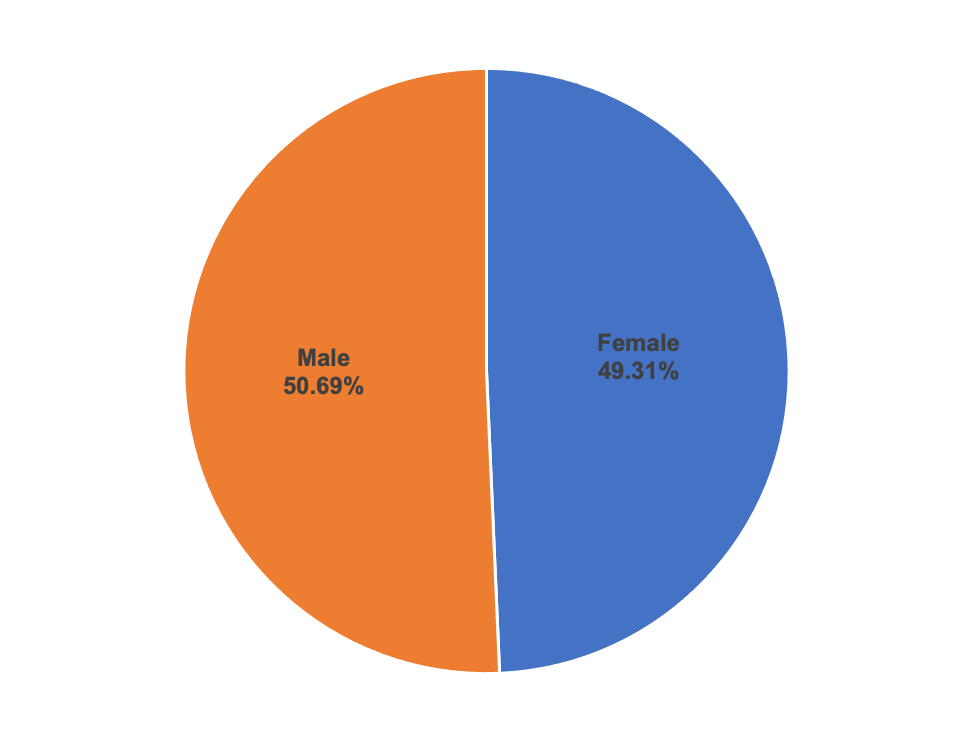


**Appendix Figure 5.** The demographic composition of cannabis users on Twitter.
